# Special Issue “The Role of Non-Coding RNAs Involved in Cardiovascular Diseases and Cellular Communication”

**DOI:** 10.3390/ijms25116034

**Published:** 2024-05-30

**Authors:** Montserrat Climent, José Luis García-Giménez

**Affiliations:** 1 Department of Biomedical Sciences, Humanitas University, 20072 Pieve Emanuele, Italy; 2 IRCCS Humanitas Research Hospital, 20089 Rozzano, Italy; 3 Department of Physiology, Faculty of Medicine and Dentistry, University of Valencia, 46010 Valencia, Spain; j.luis.garcia@uv.es; 4 Health Research Institute INCLIVA, 46010 Valencia, Spain; 5 Center for Biomedical Research Network on Rare Diseases (CIBERER), Carlos III Health Institute, 46010 Valencia, Spain

## 1. Introduction

Despite the great progress in diagnosis, prevention, and treatment, cardiovascular diseases (CVDs) are still the most prominent cause of death worldwide [1]. Atherosclerosis, myocardial infarction, cardiac hypertrophy, and heart failure are some of the most threatening CVDs [2].

Recently, we discovered that CVDs not only depend on established risk factors, such hypertension or diabetes, but are also linked to genetic variants [3,4,5]. However, the combination of genetic variants with different risk factors are not enough to explain the deep penetrance of pathologies associated with CVDs. Therefore, other mechanisms might help to better understand the overall cardiovascular-related pathologies. The contribution of epigenetic regulation in different diseases, including CVDs, is clearly increasing our knowledge about the factors involved through pathophysiological development and disease progression [2].

Epigenetic regulations include not only DNA methylation and post-translational modifications in histones but also non-coding RNAs (ncRNAs) [2,6,7]. Indeed, the involvement of ncRNAs within CVDs is well recognized as important key regulators by controlling different biological processes with a direct impact on cardiovascular functions, as well as contributing to communication between cells in the cardiovascular system and other organs [8]. So, ncRNAs are considered promising candidates for diagnosis, prognosis, disease monitoring and therapeutic purposes [9]. A common size classification of ncRNAs divides them into small ncRNAs, <200 nucleotides, and long ncRNAs, >200 nucleotides [10]. Among small ncRNAs, microRNAs (miRNAs) are capable of regulating many mRNA targets, with a direct involvement in a wide range of pathways. LncRNAs control different features of cell differentiation and development [11]. Interestingly, circular RNAs (circRNAs), which derive from the back-splicing of a precursor mRNA, have been included within lncRNAs [12,13] and have been involved in different CVDs, such as atherosclerosis [14], cardiomyopathy and cardiac hypertrophy, and ischemic stroke, among others [15].

In vascular pathologies, which with further complications can lead to heart failure, cellular remodeling is characterized by transcriptional alterations affecting the different cell populations of the cardiovascular system, including endothelial cells (ECs), vascular smooth muscle cells (VSMCs), cardiomyocytes (CMs), fibroblasts, and immune cells [16].

Cellular communication is key for proper organ homeostasis, and different ways of crosstalk [17,18,19] and different communication molecules have been discovered to be critical also for the cardiovascular system [20,21]. A plethora of studies demonstrated the importance of ncRNAs in the cardiovascular system and their involvement in cell-to-cell communication [8,22]. In fact, it is now well known that ncRNAs are considered important communication molecules due to their potential to modulate gene expression at intra- and extra-cellular levels, being secreted or transported to other cell types [19,23,24,25,26,27].

For instance, exosomes, the smallest kind of extracellular vesicles, are able to carry different cargos, including RNAs, proteins, and lipids, which can have an impact on the target cell biology by regulating a plethora of different functions [23,28]. Exosomes are secreted by all cells under physiological and pathological conditions and are released by all cells from the cardiovascular system [29]. Interestingly, when exosomes are formed, they not only capture material from the cell of origin but also retain membrane proteins specific from the cell of origin, allowing for the identification and study of the exosome’s origin [30].

In this Special Issue, we aimed at gathering information on many ncRNAs identified and studied in different CVDs, pinpointing their importance as biomarkers for several pathologies, their implication in cellular biology and their potential role as communication molecules in the cardiovascular system.

Important examples of small ncRNAs involved in CVDs are miR-133, which controls cardiac hypertrophy [31,32], miR-143, and miR-145, which have critical roles in vascular biology [33]. These miRNAs have been associated with CVDs and were identified as communication molecules within cells from the cardiovascular system. For instance, miR-133 has been found to be enriched in cardiac exosomes [30] and also regulated by different lncRNAs, such as *TUG1* in atherosclerosis and *MIAT* and *XIST* in myocardial infarction [34]. In addition, miR-143 and miR-145 are secreted by ECs and transferred indirectly through exosomes to vascular smooth muscle cells [35], modulating their phenotype. In contrast, VSMCs can transfer miR-143/5 directly by tunneling nanotubes [36] to ECs, and miR-143 can be transported indirectly through exosomes from VSMCs to ECs [37]. Interestingly, *circ_Lrp66* has been identified as being enriched in VSMCs acting as a natural sponge for miR-145 [14]. Therefore, these are good examples of the importance of the ncRNAs involved in CVDs and their implication in cellular communication.

Importantly, circulating ncRNAs are also considered important biomarkers in many pathological situations, including CVDs. For example, lncRNA *MALAT1*, which has been well studied in cancer, regulates cell cycle and cell migration [38]. Barbalata et al. demonstrated that circulating levels of lncRNA *MALAT1*, combined with *LIPCAR*, can be used to discriminate vulnerable coronary artery disease from stable and unstable angina patients, also correlated with hyperglycemia patients, and to predict unfavorable evolution of STEMI patients [39], thus being proposed as potential prognosis biomarkers. Overexpression of *MALAT1* has also been negatively associated with the development of cardiac problems in a mouse model of myocardial ischemia/reperfusion by modulating miR-145 [34,40].

Interestingly, *MALAT1* has also been found to have a cardio-protective role by being transferred within exosomes to cardiomyocytes. Doxycycline (Dox) is known to be the main form of anti-cancer drug-induced cardiac dysfunction, causing cardiac senescence. It has been shown how the lncRNA *MALAT1* is carried within the exosomes from mesenchymal stem cells in hypoxic conditions and is able to be transferred to cardiomyocytes, inhibiting miR-92a-3p which, in turn, leads to the activation of *ATG4a*, overall improvement of mitochondrial metabolism, and diminishing the cardiac remodeling caused by doxorubicin treatment, representing a novel clinical approach for Dox-induced cardiomyopathy [28,41].

Different ncRNAs have also been found to regulate inflammation in the cardiovascular system. The inflammatory miR-223 [42] has been found to be enriched in exosomes released from mesenchymal stem cells and being transferred to CMs, where it downregulates its targets, reducing inflammation, cell death, and overall heart failure in a model of septic-induced cardiomyopathy [23]. Inflammatory responses during sepsis lead to endothelial activation and promote the expression of cell adhesion molecules, such as Intercellular Adhesion Molecule 1 (ICAM-1), which contribute to platelet adhesion and tissue factor (TF), which initiates thrombosis, also in atherosclerotic plaques. Interestingly, miR-223 has been identified in microparticles from thrombin-activated platelets which can be internalized by ECs and mRNA targets to be modulated, such as TF or ICAM-1, regulating the coagulation cascade and atherosclerosis plaque rupture [43].

## 2. Conclusions

As demonstrated by most of the publications within this Special Issue, miR-155 has been shown to be a crucial pro-inflammatory miRNA that is involved in many ways in the cardiovascular system. Importantly, one of the most relevant miRNAs described in this Special Issue was miR-155. The upregulation of this miRNA promotes endothelial dysfunction, leading to vascular leakage. Moreover, miR-155 has been observed to be increased in mice during sepsis, as well as in plasma from patients in septic conditions, and it has been proposed as a key regulator of coagulation [43]. Furthermore, Laura Francés et al. described how miR-155 can be transferred, through macrovesicles, from neutrophils to ECs modulating its targets, such as NF-kB, implicated in a vascular inflammatory response [23]. Also, during atherosclerosis, miR-155 is able to be transferred, by exosomes, from VSMCs to ECs, controlling endothelial damage, as described by Zhang et al. [28]. Furthermore, in Barbalata et al., it was shown that miR-155 plasma levels can be used for the prognosis model of coronary artery disease, associated with the previously mentioned lncRNAs, *LIPCAR*, and *MALAT1* [39].

Importantly, the research on ncRNAs carried by exosomes and their role in cell-to-cell communication, as well as the interaction of different families of ncRNAs, such as small RNAs, circRNAs, and lncRNAs, is an important field in CVD research. The comprehension of these intriguing interactions and their impact on the physiopathology of CVDs may directly impact further clinical applications by providing new potential biomarkers and accelerating the development of new therapeutic approaches.
